# Supplementary material for: Rab1-AMPylation by Legionella DrrA is allosterically activated by Rab1
Source: Nat Commun. 2021 Jan 19;12:460. doi: 10.1038/s41467-020-20702-2 (PMC7815794; doi:10.1038/s41467-020-20702-2)
Supplement: Supplementary file 1 — Supplementary Information [file 41467_2020_20702_MOESM1_ESM.pdf]

Title

Rab1-AMPylation by Legionella DrrA is allosterically activated by Rab1

Authors

Jiqing Du[1,2], Marie-Kristin von Wrisberg[3], Burak Gulen[1,2], Matthias Stahl[2,4], Christian Pett[5], Christian Hedberg[5], Kathrin Lang[3*], Sabine Schneider[6*], Aymelt Itzen[1,2,7*]

Affiliations

[1] Center for Experimental Medicine, Institute of Biochemistry and Signal Transduction, Universitätsklinikum Hamburg-Eppendorf (UKE), Hamburg 20246, Germany
[2] Center for Integrated Protein Science Munich (CIPSM), Department of Chemistry, Technical University of Munich, Garching, 85748, Germany
[3] Center for Integrated Protein Science Munich (CIPSM), Department of Chemistry, Technical University of Munich, Institute for Advanced Study, Garching, 85748, Germany
[4] Present address: Science for Life Laboratory, Department of Oncology-Pathology, Karolinska Institutet, Box 1031, 171 21 Solna, Stockholm, Sweden
[5] Chemical Biology Center (KBC), Department of Chemistry, Umeå University, Linnaeus väg 10, 90187, Umeå, Sweden.
[6] Center for Integrated Protein Science Munich (CIPSM), Department of Chemistry, Ludwig-Maximilians-University Munich, München, 81377, Germany
[7] Center for Structural Systems Biology (CSSB), University Medical Centre Hamburg-Eppendorf (UKE), Hamburg, Germany.

These authors contributed equally: Jiqing Du, Marie-Kristin von Wrisberg

*For correspondence: Kathrin Lang (kathrin.lang@tum.de), Sabine Schneider (sabine.schneider@cup.lmu.de), Aymelt Itzen (a.itzen@uke.de)

Supplementary Figures

Supplementary Figure 1

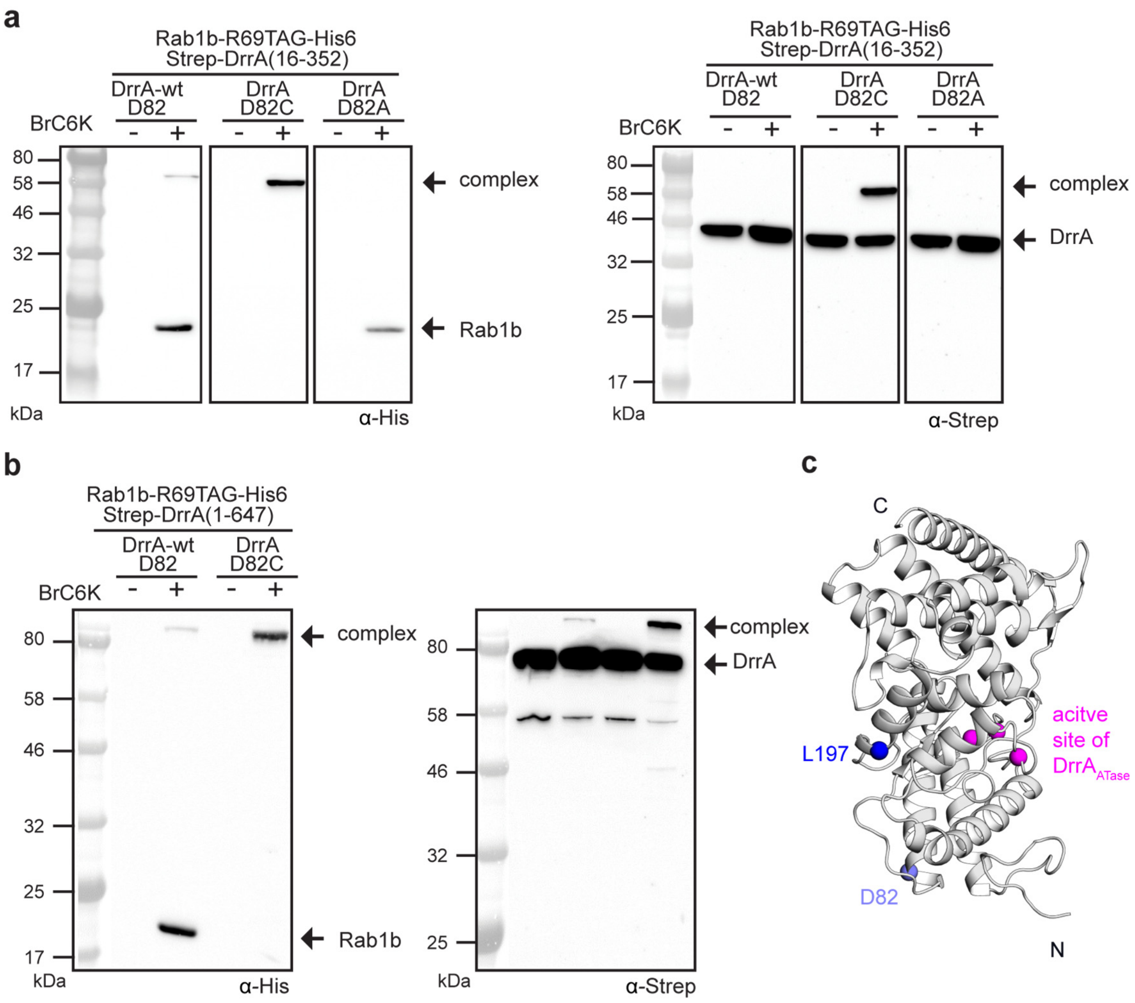

Supplementary Figure 1. Covalently crosslinking the Rab1b-DrrA$_{ATase}$ interface using UAAs. **(a)** *In vivo* crosslink formation between R69BrC6K$_{Rab1b}$ and wild type DrrA$_{16\text{-}352}$ and DrrA$_{16\text{-}352}$ -D82C. Introduction of an Ala mutation at position D82$_{DrrA}$ abolishes crosslinking with R69BrC6K$_{Rab1b}$. **(b)** Crosslinking between R69BrC6K$_{Rab1b}$ and full-length DrrA$_{1\text{-}647}$ mutants. **(c)** Cartoon representation of the DrrA-ATase domain from our DrrA:Rab8a-complex structure. Positions of L197$_{DrrA}$ (color blue) and D82$_{DrrA}$ (color light blue) are indicated. Pink spheres: the active site of DrrA$_{ATase}$. Source data are provided as a Source Data file.

Supplementary Figure 2

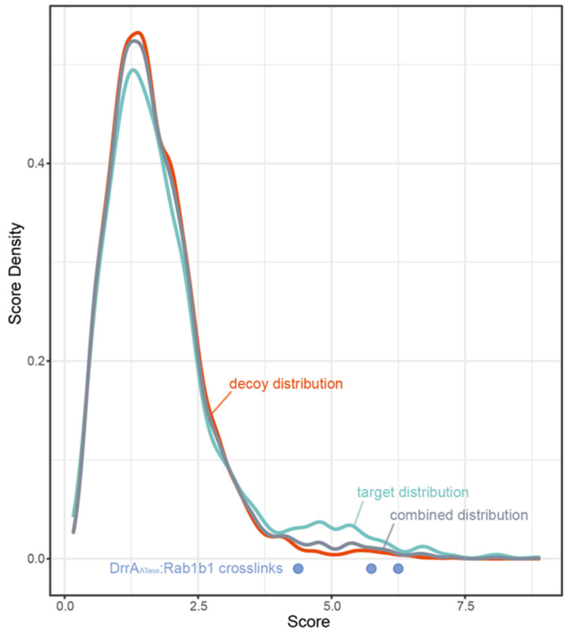

Supplementary Figure 2. The scores of all recorded crosslink identifications were collected and their density was modeled across the whole range (gray curve). In order to evaluate the confidence in the three DrrA:Rab1b crosslink spectra (filled blue circles), the scores were classified as target scores (identifications with target sequences only) and decoy scores (identifications involving decoy peptides, i.e. reverse sequences). Notably, an identification was counted as a decoy one, when one of the partner peptides could be assigned to a decoy protein. For each of the classes, target and decoy, a distinct density distribution was created. Turquois: target score distribution. Red: decoy score distribution. The DrrA:Rab1b crosslink spectra exhibit scores which fall in a range where the target distribution is outperforming the decoy distribution indicating a good credibility of these measurements.

Supplementary Figure 3

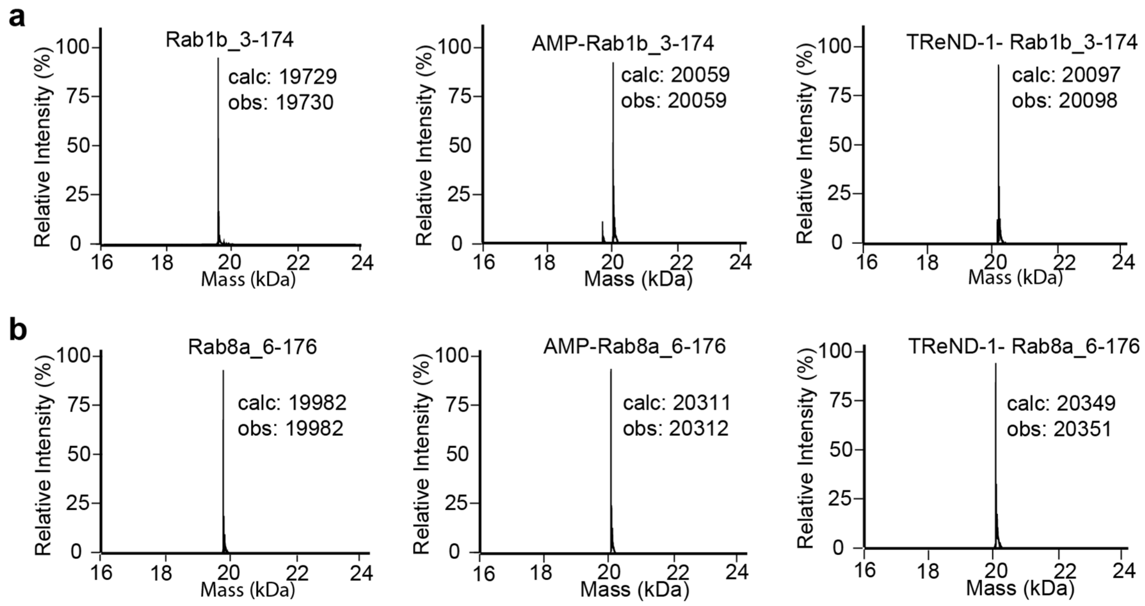

Supplementary Figure 3. Intact mass spectrometry analysis of Rab1b/8a and modified Rab1b/8a. TReND-1 is transferred by $DrrA_{16-352}$ to $Rab1b_{3-174}$ and $Rab8a_{6-176}$. **(a)** *In vitro* modification of Rab1b with ATP or TReND-1 by $DrrA_{16-352}$. **(b)** *In vitro* modification of Rab8a with ATP or TReND-1 by $DrrA_{16-352}$. Source data are provided as a Source Data file.

Supplementary Figure 4

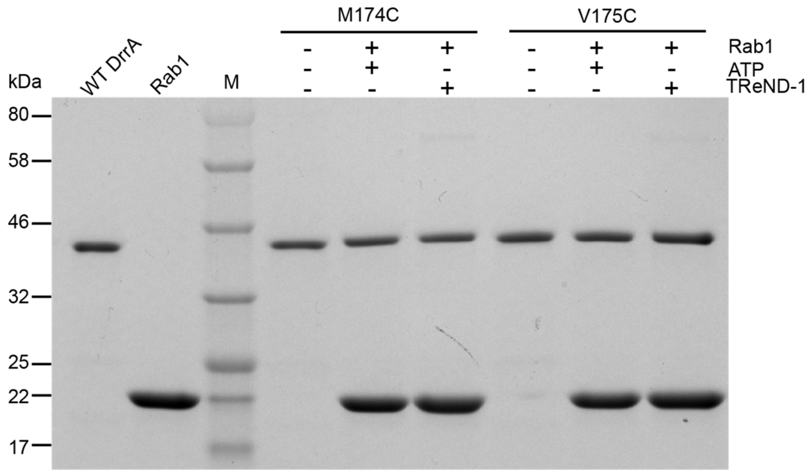

Supplementary Figure 4. SDS-PAGE shift analysis of DrrA:TReND:Rab1 complex formation. Residues in the vicinity of the ATase's active site ($D110_{DrrA}$, $D112_{DrrA}$, and $D150_{DrrA}$) were further selected for testing DrrA:TReND:Rab1 complex formation. Source data are provided as a Source Data file.

Supplementary Figure 5

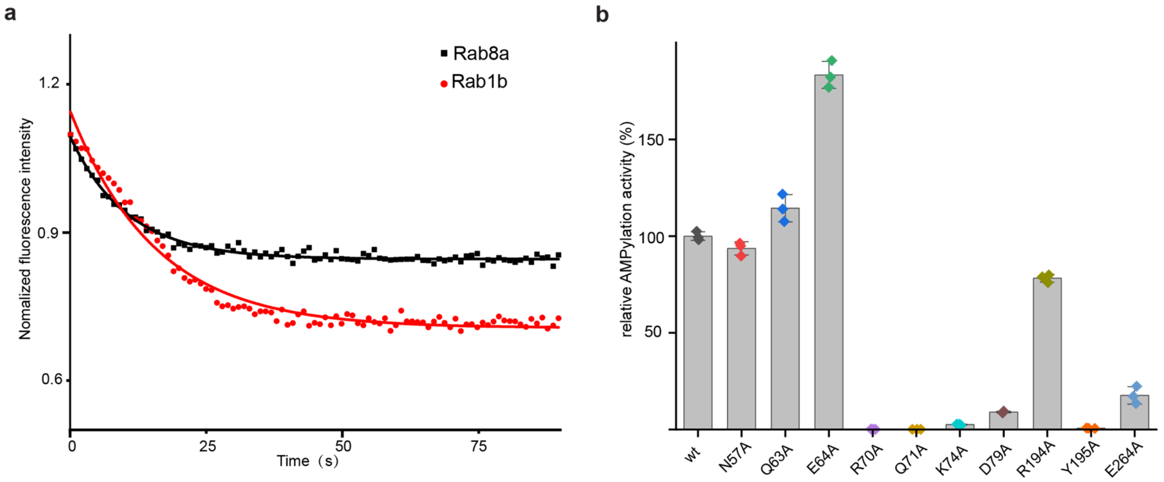

Supplementary Figure 5. DrrA mediated Rab8a AMPylation. **(a)** Catalysis kinetics of DrrA toward Rab1b:GppNHp and Rab8a:GppNHp. The k_{cat}/K_M value of wt $DrrA_{16\text{-}352}$ toward Rab8a:GppNHp is 9.0×10^5 M^{-1} s^{-1} ($\pm$ 1.2×10^5 M^{-1} s^{-1}). The k_{cat}/K_M value of wt $DrrA_{16\text{-}352}$ toward Rab1b is 6.2×10^5 M^{-1} s^{-1} ($\pm$ 2.0×10^4 M^{-1} s^{-1}). Data are means ± standard error of the mean (SEM) from three independent experiments. The tryptophan fluorescence intensity before adding DrrA is normalized to 1.0. **(b)** Catalysis kinetics of DrrA mutants toward Rab8a: GppNHp. The k_{cat}/K_M value of wt $DrrA_{16\text{-}352}$ is 9.0×10^5 M^{-1} s^{-1} ($\pm$ 1.2×10^5 M^{-1} s^{-1}). Data are means ± standard error of the mean (SEM) from three independent experiments. Source data are provided as a Source Data file.

Supplementary Figure 6

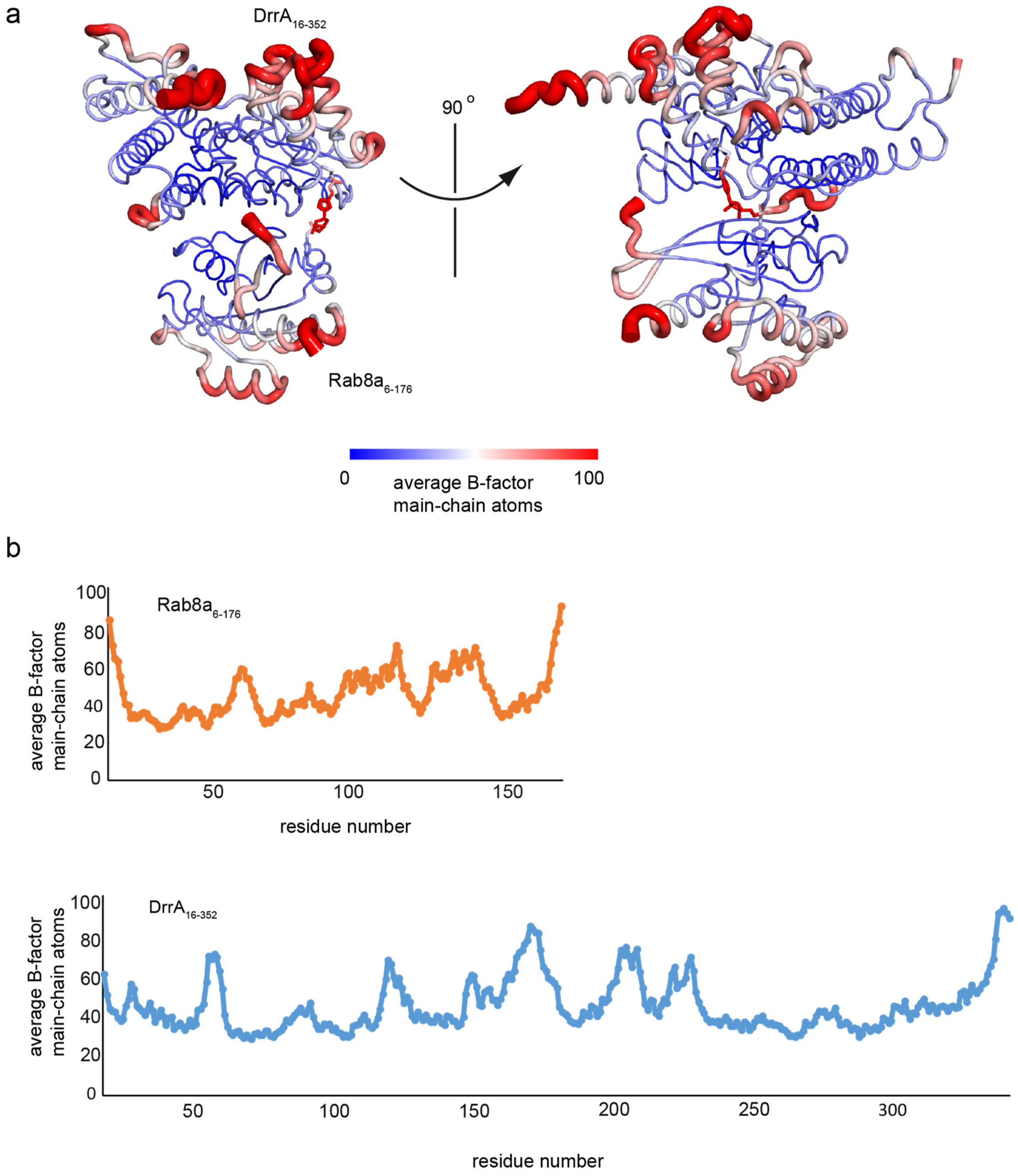

Supplementary Figure 6. Temperature factor analysis of the DrrA$_{16\text{-}352}$-L197C-TReND1-Rab8a$_{6\text{-}176}$ complex. **(a)** Complex structure with B-factors of the main chain atoms mapped onto the ribbon diagram. The TReND cross linker is shown as stick model. **(b)** Plot of the temperature factors per residue generated with Baverage[1]. (Average B-factor of the TReND cross linker = 80.4).

Supplementary Figure 7

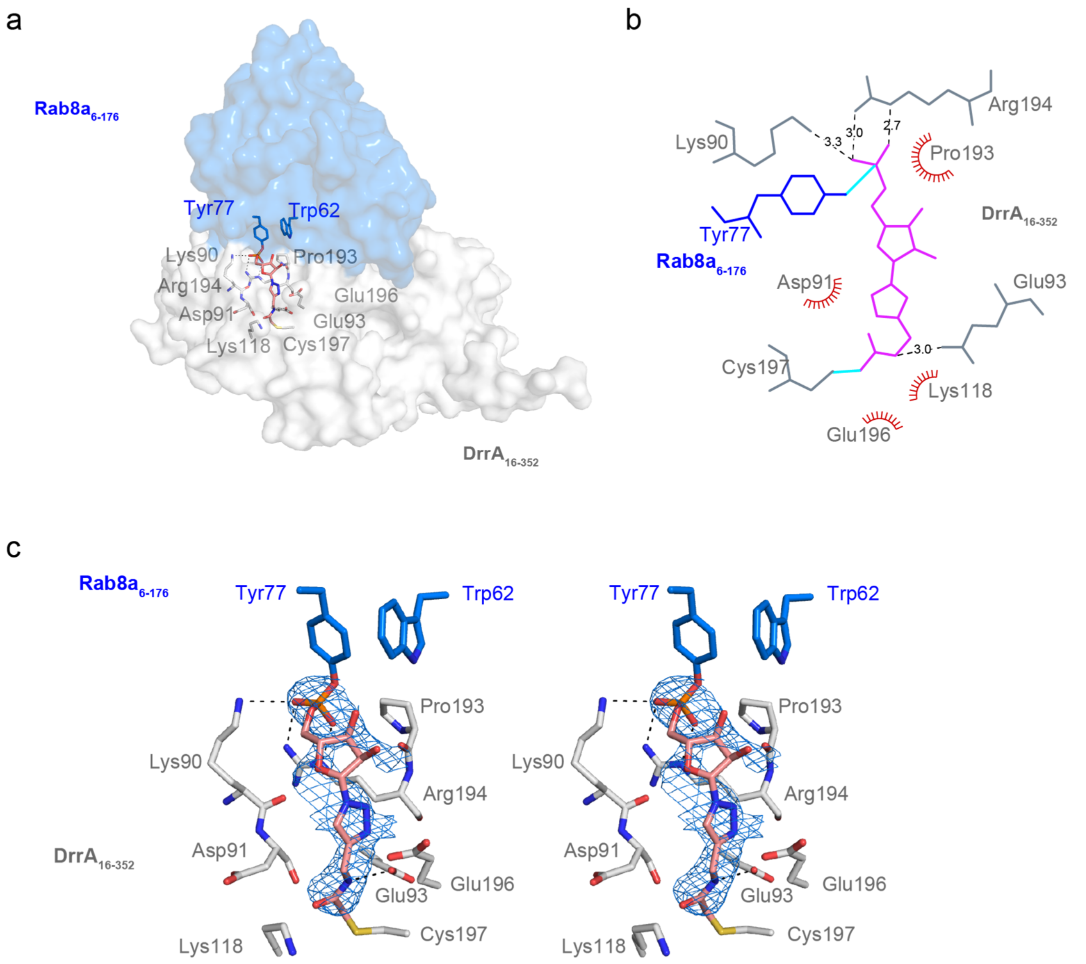

Supplementary Figure 7. TReND-linker binding site. **(a)** Surface representation of the DrrA:Rab8a complex, with the TReND-linker and surrounding residues shown as stick models. **(b)** Ligplot diagram [2] of the amino acid residues interacting with the TReND linker. Hydrogen bonds are drawn as black lines, residues in Van der Waals- distance are highlighted as red half circles and the covalent bonds are shown in cyan. **(c)** Stereo view of the TReND-linker (pink) and its Fo-Fc simulated annealing omit-electron density map (blue mesh, contoured at 2σ) and surrounding amino acids. (DrrA: grey; Rab8: blue, TReND-linker: pink)

Supplementary Figure 8

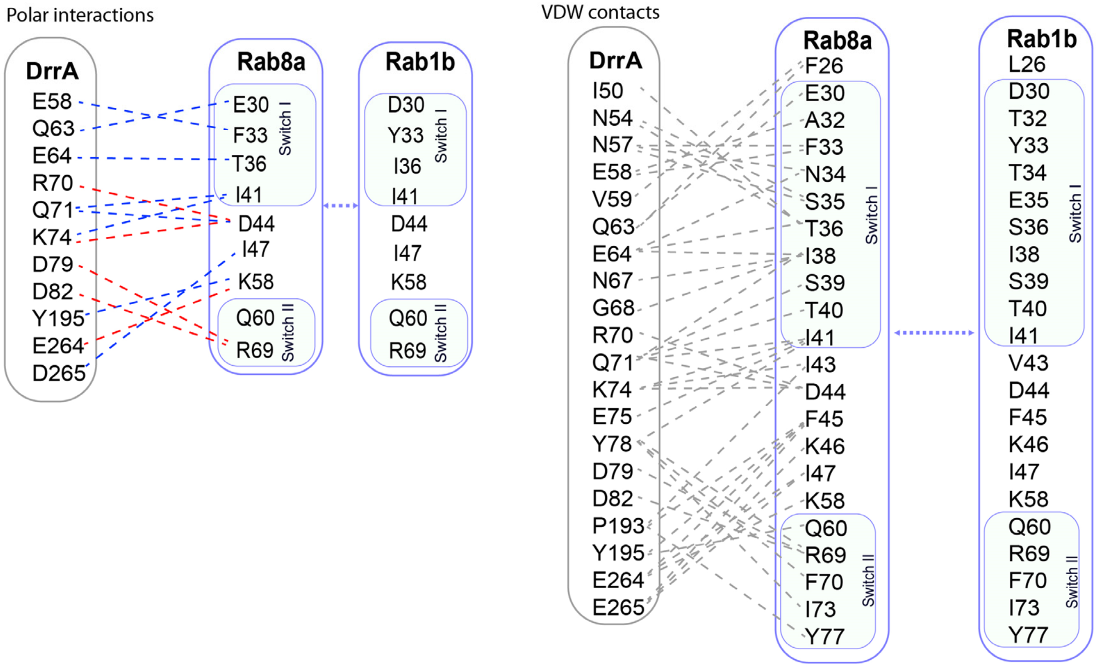

Supplementary Figure 8. Polar interactions and VDW contacts in DrrA:Rab8a complex. Polar interactions are shown with dashed lines: hydrogen bonds (blue), salt-bridges (red). VDW contacts are shown with grey dashed lines. The corresponding interaction residues in Rab1 are shown close to the Rab8a panels.

Supplementary Figure 9

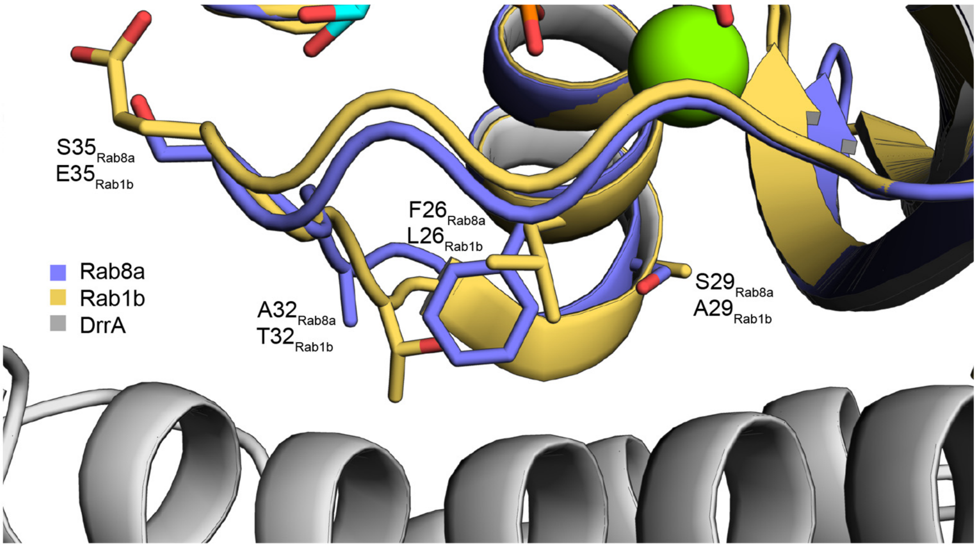

Supplementary Figure 9. Amino acid differences between Rab1b and Rab8a in the Rab-DrrA-interface. Superimposition of Rab8a with Rab1b in the Rab8a:DrrA-complex. Rab1b amino acid side chains that differ from Rab8a do not cause structural clashes, explaining why both Rabs are AMPylated by $DrrA_{16\text{-}352}$ *in vitro*.

Supplementary Figure 10

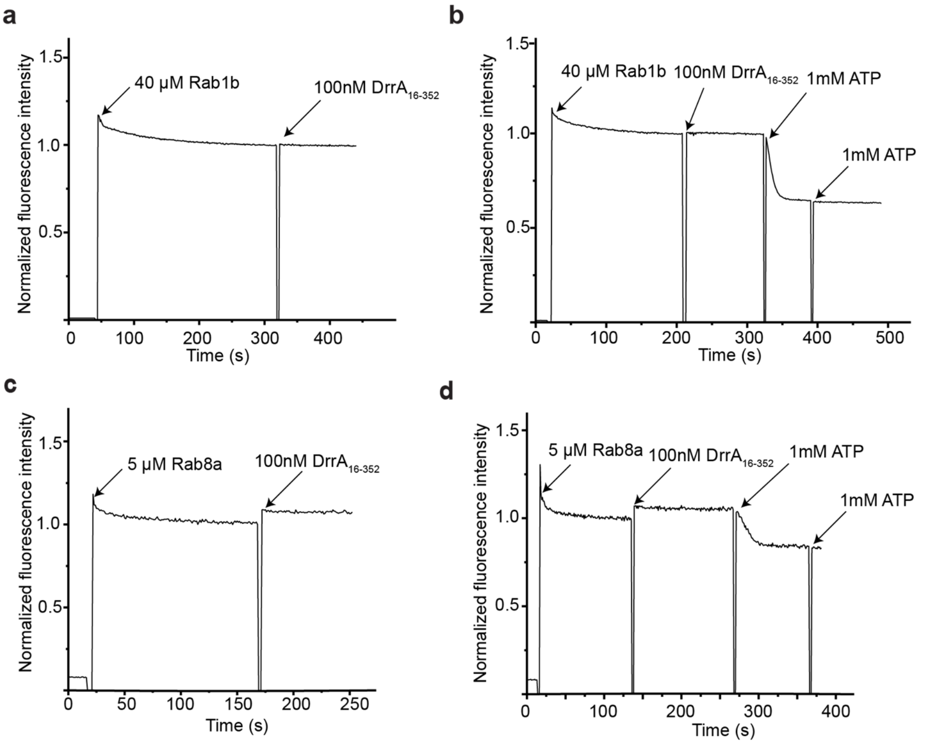

Supplementary Figure 10. Representative fluorescence traces and controls for the kinetics of Rab-AMPylation. (a) Negative control for enzyme kinetics of DrrA mediated Rab1b:GppNHp AMPylation based on time-resolved tryptophan fluorescence. **(b)** Positive control for enzyme kinetics of DrrA mediated Rab1b:GppNHp AMPylation based on time-resolved tryptophan fluorescence. **(c)** Negative control for enzyme kinetics of DrrA mediated Rab8a:GppNHp AMPylation based on time-resolved tryptophan fluorescence. **(d)** Positive control for enzyme kinetics of DrrA mediated Rab8a:GppNHp AMPylation based on time-resolved tryptophan fluorescence. The tryptophan fluorescence intensity of Rab proteins are normalized to 1.0. All panels: $Rab1b_{3-174}$, $Rab8a_{6-176}$, and $DrrA_{16-352}$ are referred to as Rab1b, Rab8a and DrrA, respectively. Source data are provided as a Source Data file.

Supplementary Figure 11

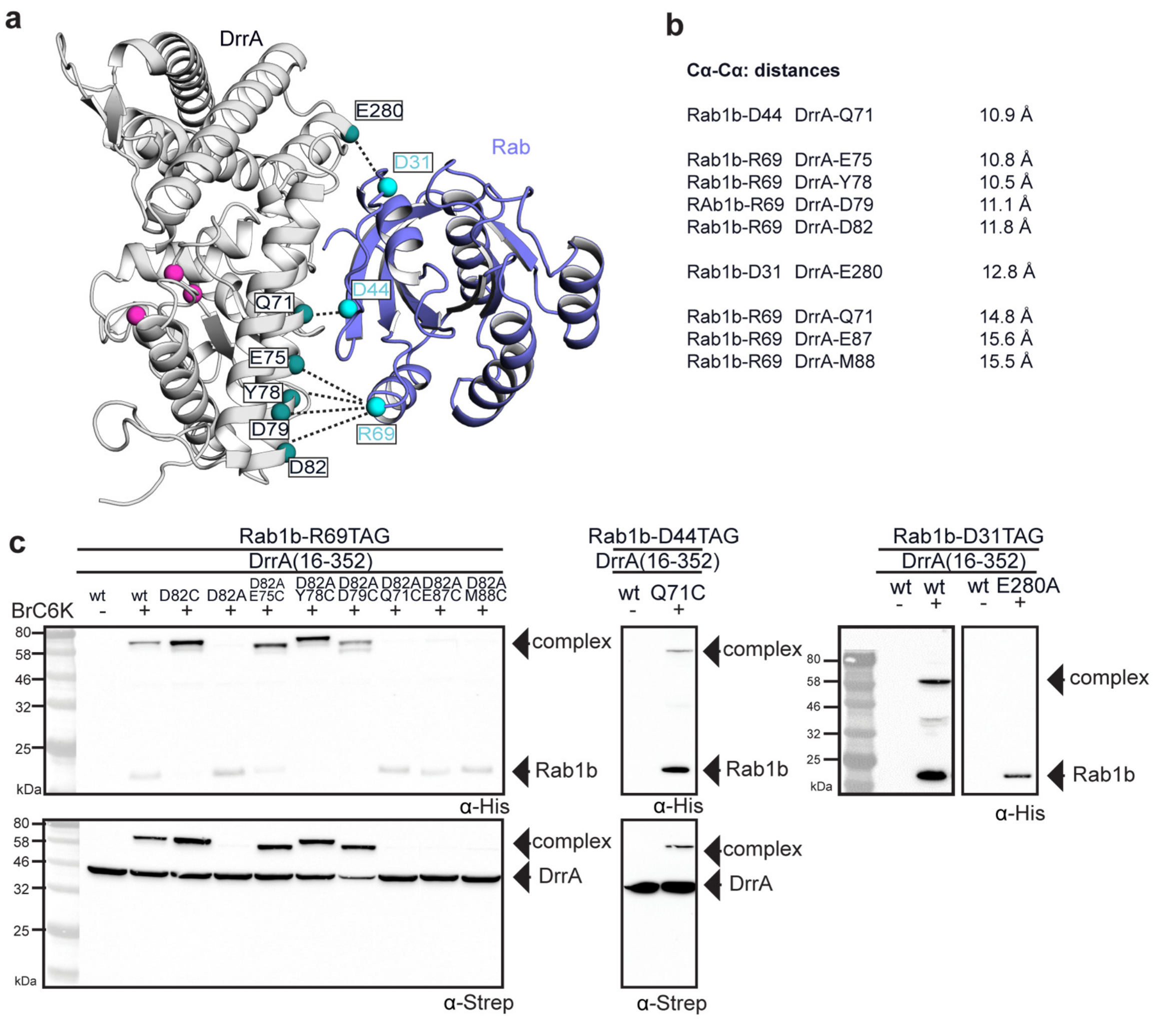

Supplementary Figure 11. Crosslinking assays guided by the covalent DrrA:Rab8a complex structure. **(a)** Cartoon depiction of selected interactions for *in cellulo* crosslinking in *E. coli*. Pink spheres are the catalytic Asp residues of DrrA. **(b)** Representation of Cα-Cα distances from selected interactions on the DrrA:TReND:Rab complex structure. **(c)** Validating the NC Rab-DrrA$_{\mathrm{ATase}}$ interface by co-expression pairwise BrC6K$_{\mathrm{Rab1}}$ and Cys$_{\mathrm{DrrA}}$ mutations in *E. coli*. Covalent crosslink formations were confirmed by α-His6 and α-Strep Western blotting. Source data are provided as a Source Data file.

Supplementary Figure 12

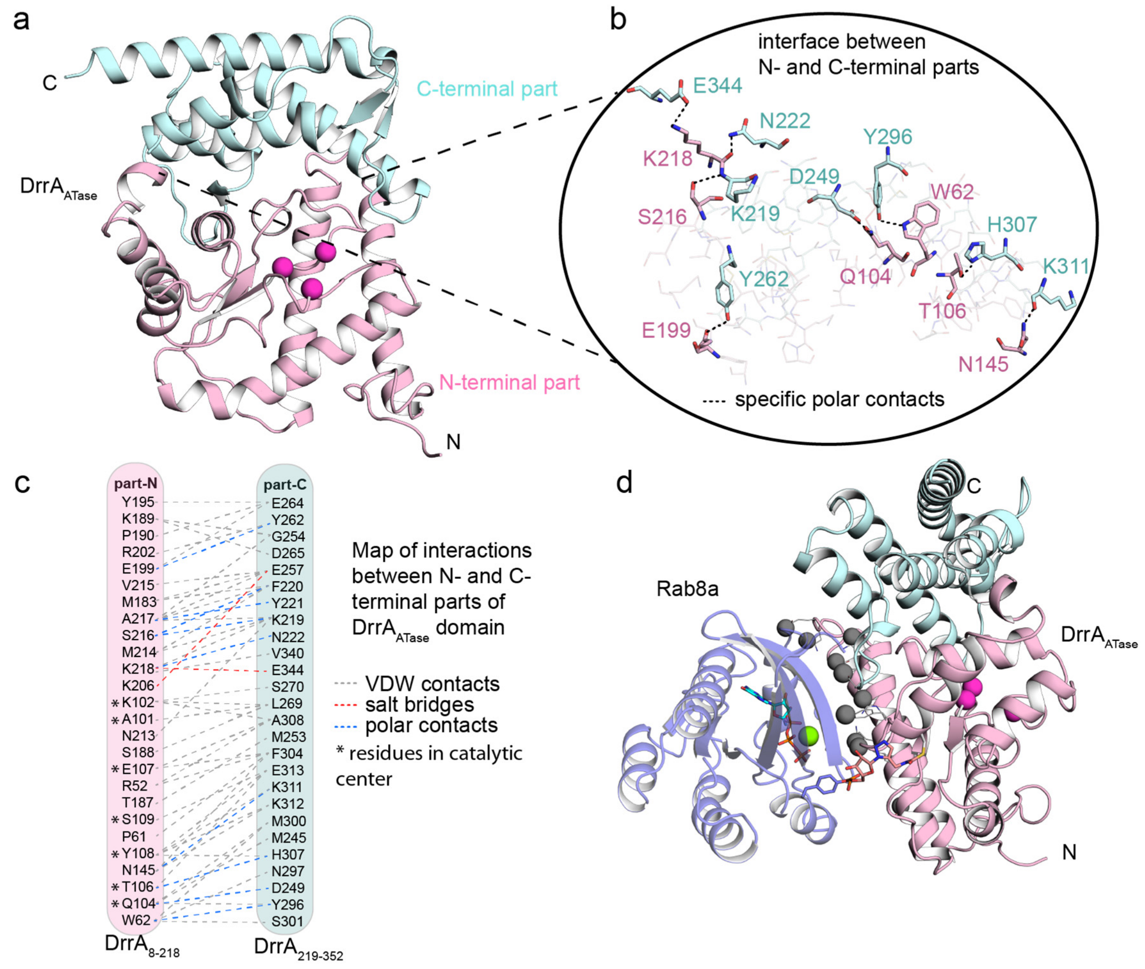

Supplementary Figure 12. Intra- and intermolecular interactions of N- and C-terminal parts of the DrrA ATase domain. **(a)** Schematic representation of N- terminal part (part-N, DrrA8-218, color pink) and C- terminal part (part-C, DrrA$_{218-340}$, color cyan) in DrrA$_{16-352}$. **(b)** Specific polar interaction involving sidechain-sidechain or sidechain-backbone between part-N (pink) and part-C (cyan). Interface between the parts is shown in transparent background. **(c)** Interaction map of part-N and part-C of DrrAATase domain. The residues within the catalytic site are illustrated with asterisks. Van der Waals contacts are gray, polar contacts are blue, salt bridges are red. d) Mainly the residues in the part-N contacts Rab1 in the back site and only residue which contacts Rab1 in the back site from part-C is E264. Interacting residues are highlighted with black sphere.

Supplementary Figure 13

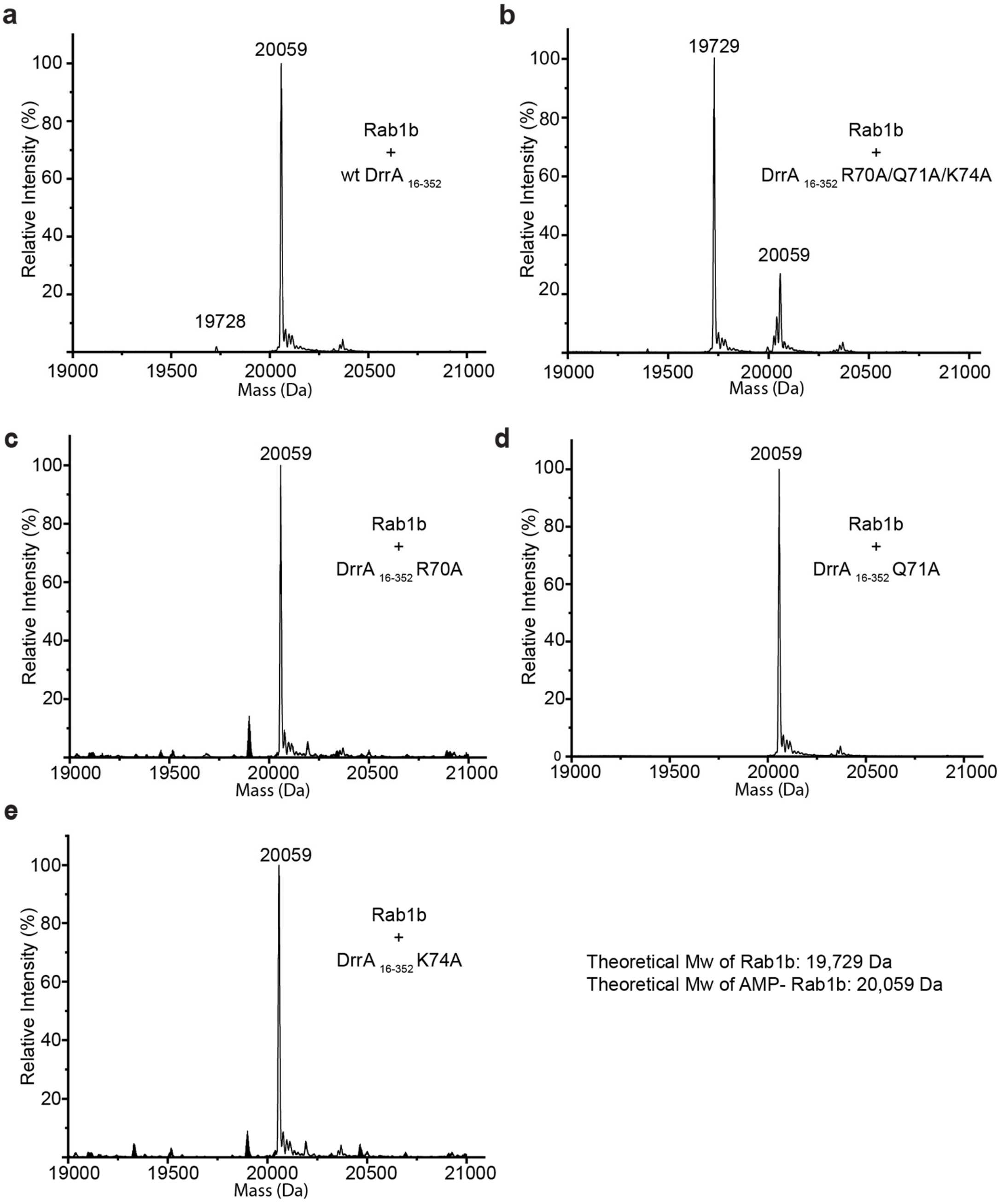

Supplementary Figure 13. *In vitro* AMPylation of 100 µM Rab1b$_{3\text{-}174}$:GppNHp by DrrA$_{16\text{-}352}$ (5 µM) and DrrA NC-RBS-mutants (5 µM). AMPylation was carried out for 72 h. The mass of AMPylated and non-AMPylated Rab1b$_{3\text{-}174}$ was determined by intact mass spectrometry. The triple-mutant R70A/Q71A/K74A has minor AMPylation activity, whereas the single mutants are still capable of producing AMPylated Rab1b (Mw(Rab1b$_{3\text{-}174}$)= 19,729 Da, Mw(AMP-Rab1b$_{3\text{-}174}$)=20,059 Da. **(a)** Rab1b AMPylated by wt DrrA$_{16\text{-}352}$. **(b)** Rab1b AMPylated by DrrA$_{16\text{-}352}$R70A/Q71A/K74A. **(c)** Rab1b AMPylated by DrrA$_{16\text{-}352}$R70A. **(d)** Rab1b AMPylated by DrrA$_{16\text{-}352}$Q71A. **(e)** Rab1b AMPylated by DrrA$_{16\text{-}352}$K74A. Source data are provided as a Source Data file.

Supplementary Figure 14

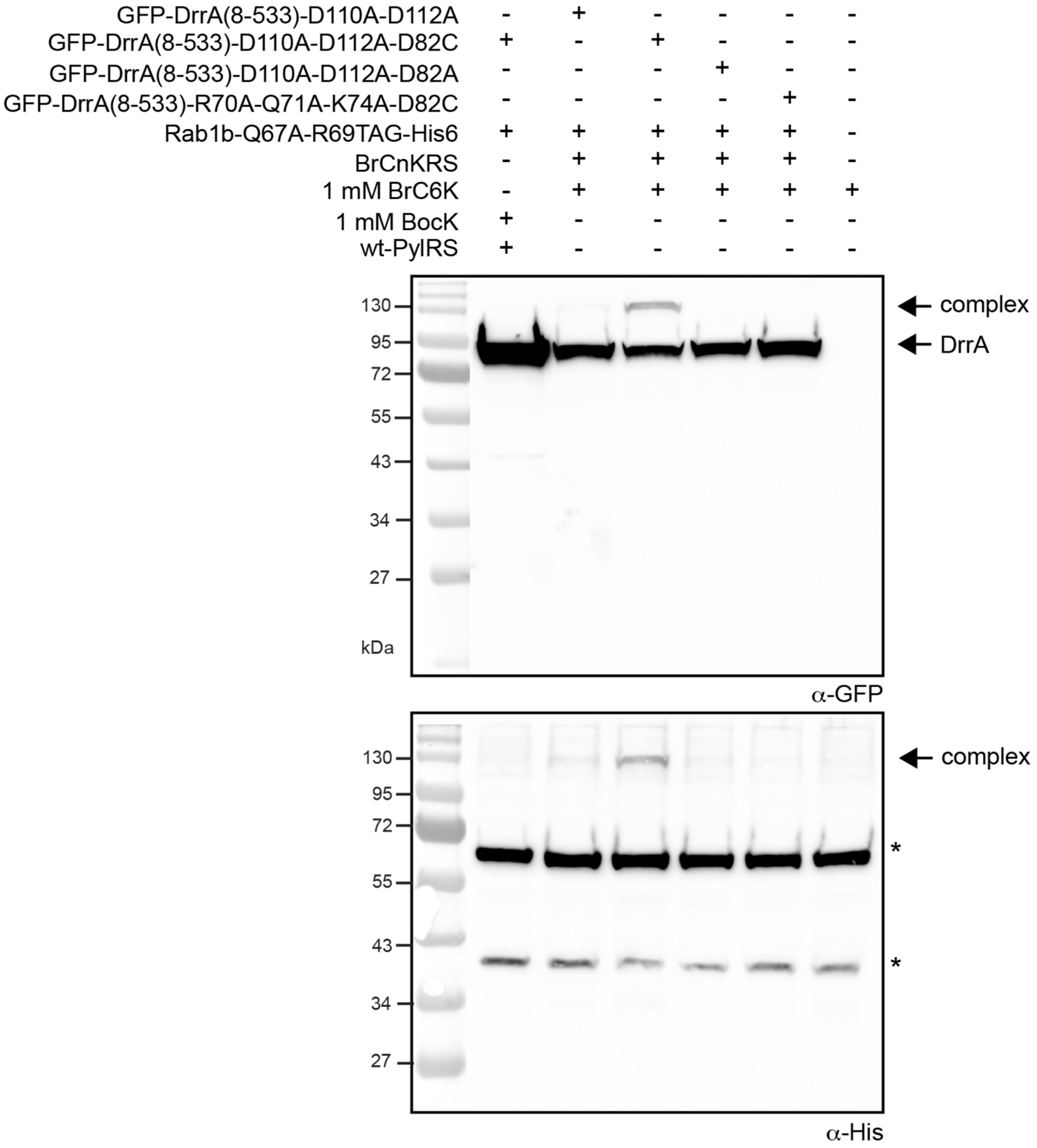

Supplementary Figure 14. Proximity-triggered crosslinking in living mammalian cells. The indicated eGFP-DrrA variants were co-expressed with BrC6K- or BocK-bearing Rab1b in HEK293T cells. Covalent crosslink formations were confirmed by α-His6 and α-GFP Western blotting. BocK indicates Nε-tert-butoxycarbonyl-L-lysine, a non-crosslinking competent UAA that is well incorporated by the wt-PylRS. With asterisk indicated bands stem from His-bearing endogenous proteins arising also in non-transfected cells. Source data are provided as a Source Data file.

Supplementary Figure 15

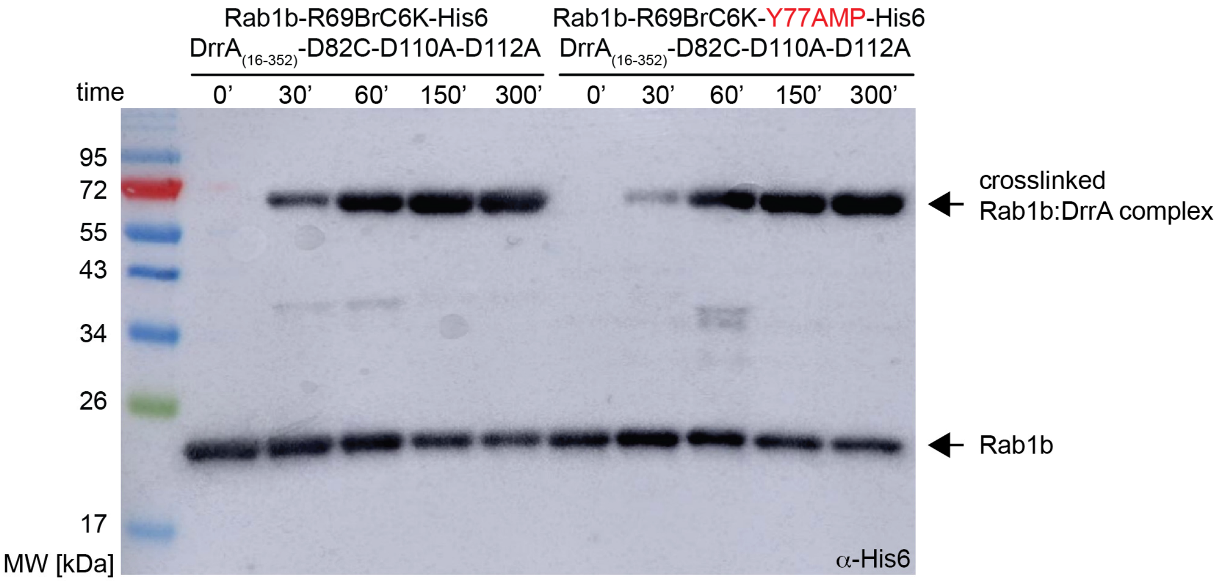

Supplementary Figure 15. Covalent complex formation between Rab1b-R69BrC6K-His6 and DrrA-D82C does not depend on AMPylation status of Rab1b. *In vitro* crosslinking of non-AMPylated or AMPylated Rab1b variants (Rab1b$_{3-174}$-Q67A-R69BrC6K-His6 or Rab1b$_{3-174}$-Q67A-R69BrC6K-Y77AMP-His6) with an AMPylation deficient DrrA variant (DrrA$_{16-352}$-D82C-D110A-D112A) shows similar formation of covalently crosslinked Rab1b:DrrA complex. 15 µM of the corresponding Rab1b variant were mixed with 10 µM DrrA variant and reactions were quenched at the indicated time points and analyzed by SDS-PAGE and α-His6 western-blot. Source data are provided as a Source Data file.

Supplementary Figure 16

a

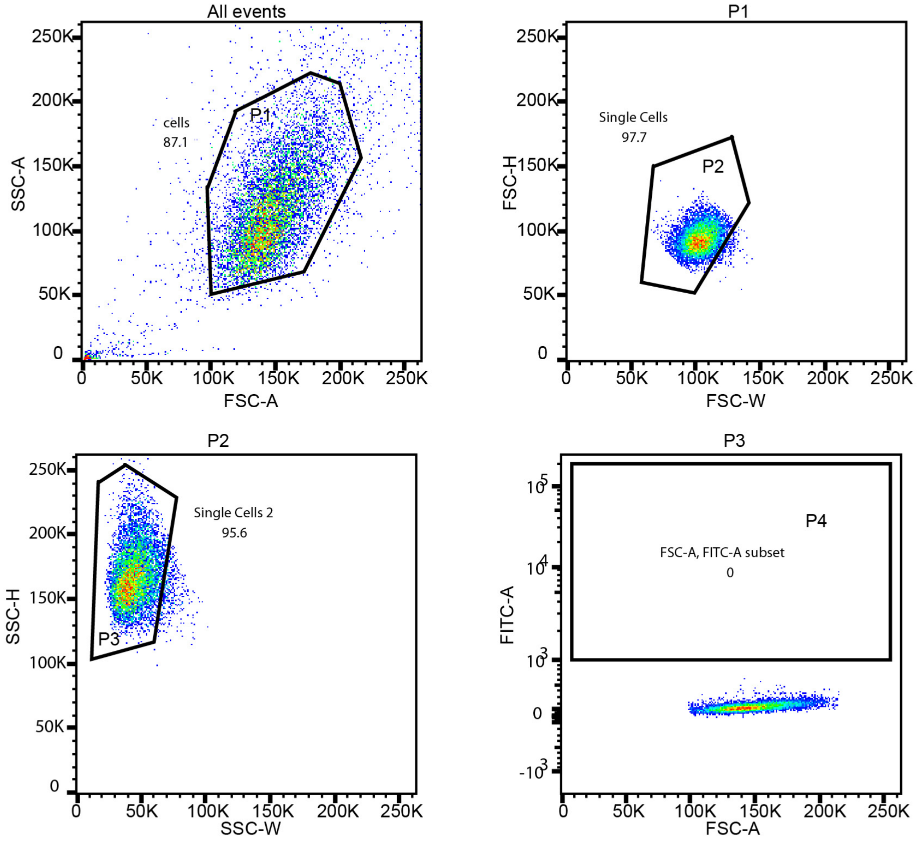

b

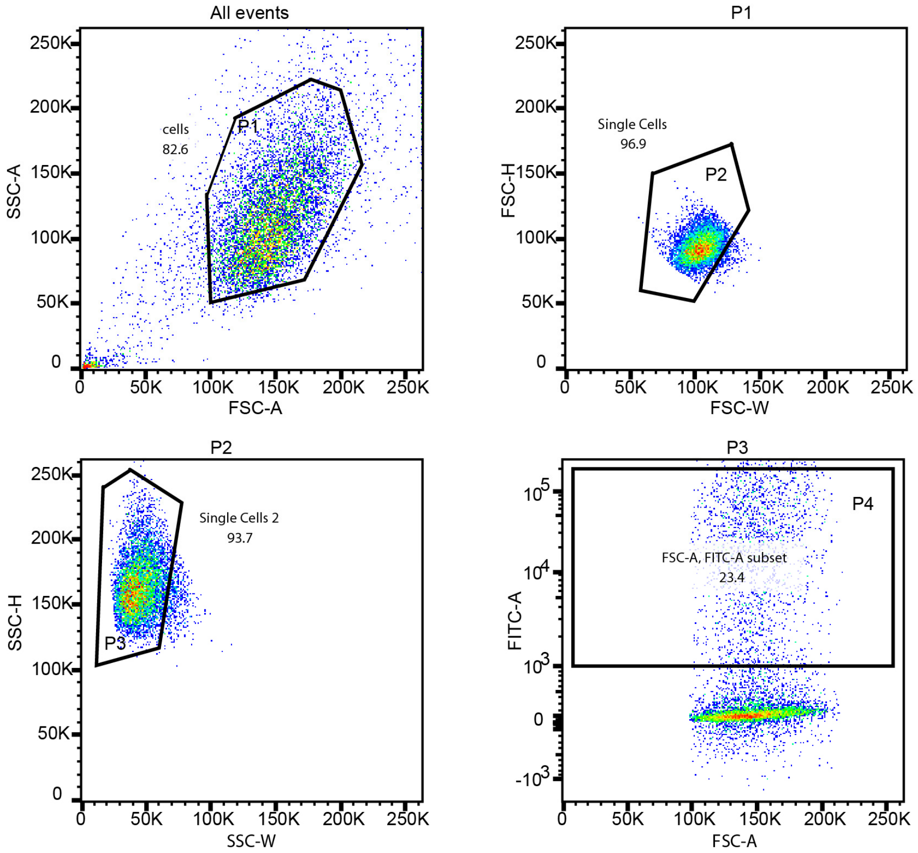

Supplementary Figure 16. Representation of the gating strategy for sorting GFP- positive H1299 cells. Side scatter area (SSC-A) versus forward scatter area (FSC-A) density plot was applied for excluding debris and dead H1299 cells. Selected population was named as P1. The plot (forward scatter height (FSC-H) versus forward scatter width (FSC-W)) is used to discriminate doublets from single cells; selected population was marked as P2. The plot (side scatter height (SSC-H) versus side scatter width (SSC-W)) was further used to ensure the selection of single cells, selected population was labelled as P3. Subsequently, the last plot (FITC-A (GFP channel) versus FSC-A) was applied to separate the GFP- positive cells (P4 population) from the GFP- negative cells. The proper gating was set up using non-transfected cells as negative control. **(a)** Non-transfected H1299 cells were taken as negative control. **(b)** GFP- transfected H1299 cells. Source data are provided as a Source Data file.

Supplementary Figure 17

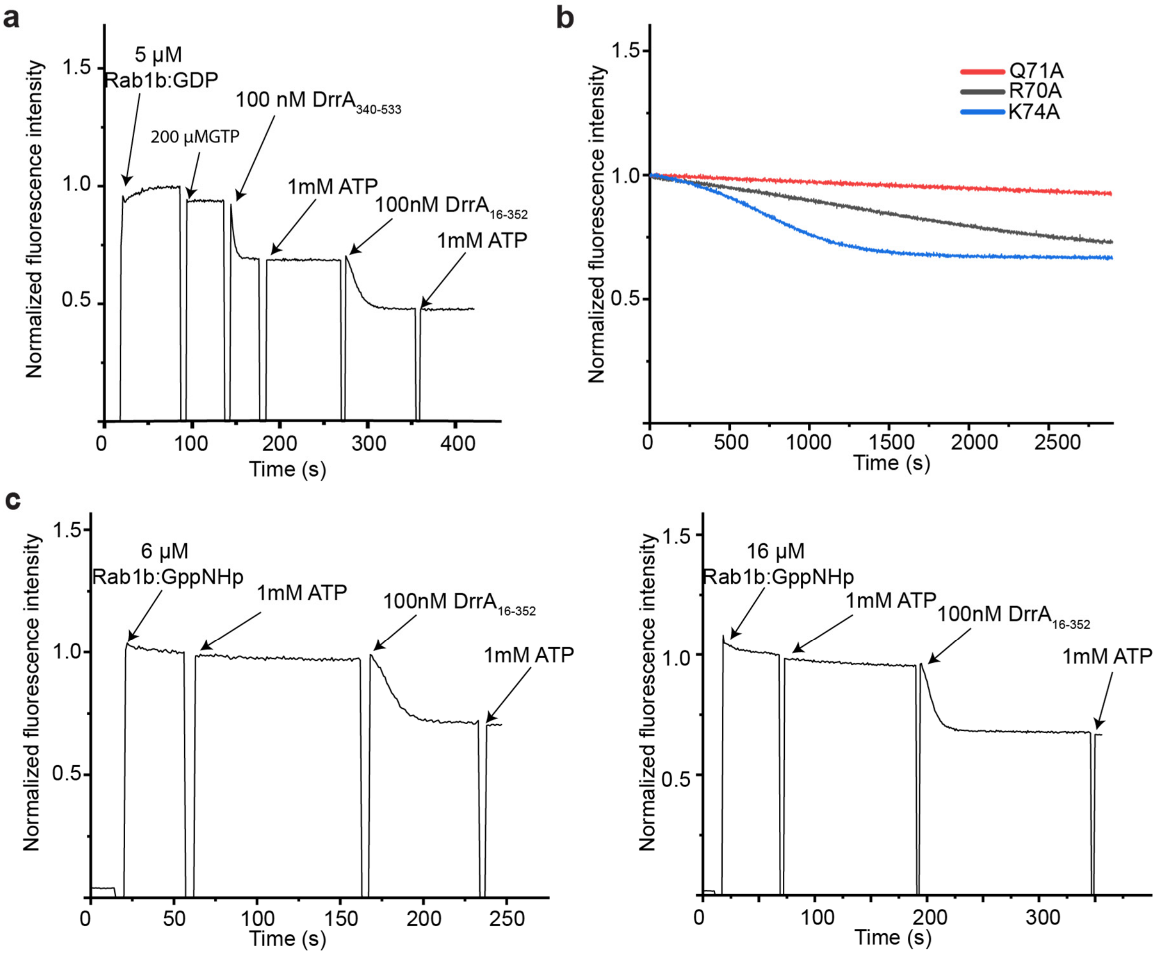

Supplementary Figure 17. Representative kinetics profiles for DrrA- catalyzed AMPylation of Rab1b by time- resolved tryptophan fluorescence. **(a)** Standard procedure for studying the AMPylation activity of wt DrrA or DrrA mutants. **(b)** Catalysis kinetics of DrrA mutants (R70A, Q71A, and K74A) toward Rab1b:GTP. The tryptophan fluorescence intensity is normalized to 1.0 before adding DrrA. **(c)** Representative kinetic profiles for allosteric activation. The tryptophan fluorescence intensity of Rab1b:GppNHp is normalized to 1.0.

Supplementary Tables

Supplementary Table 1

Supplementary Table 1. Identified crosslink peptides between DrrAATase and Rab1b-R69BrC6K. Crosslinks were identified and scored using the software Kojak [3, 4]. The site of the crosslink is indicated in red with the corresponding amino acid numbering next to it. Kojak software identified an ester crosslink between D82 in DrrA and BrC6K at position 69 in Rab1b. This was corroborated by biochemical experiments with D82A (no crosslink formation) and D82C (more efficient thioether crosslink formation) mutations. With a lower score also a third crosslinked peptide was identified, indicating an ester crosslink formation between BrC6K at position 69 in Rab1b and E38 in DrrA. This could not be verified by biochemical analysis.

Scan Number	***Protein 1***	***Protein 2***	***Crosslink Peptide (bold crosslink site indicated by positions for each peptide partner)***	***Score***	***Charge***	***Theo./ Exp. Mass (Da)***	***Mass error (ppm)***
29099	DrrA	Rab1b	-.EMIYDAF**D**82ALGVEMPKDMEVHFAGSLAK(8)—LQIWDTAGAE(**BrC6K**)69FR(11).-	6.25	+5	4743.306079<br>4743.315433	-<br>1.9720
29082	DrrA	Rab1b	-.EMIYDAF**D**82ALGVEMPKDMEVHFAGSLAK(8)--LQIWDTAGAE(**BrC6K**)69FR(11).-	5.745	+5	4743.306079<br>4743.315173	-<br>1.9173
14755	Rab1b	DrrA	-.LQIWDTAGAE(**BrC6K**)69FR(11)—KF**E**38EYQNK(3).-	4.375	+4	2714.370280<br>2714.372266	-<br>0.7317

Supplementary Table 2

Supplementary Table 2. Data collection and refinement statistics.

	**$DrrA_{16\text{-}352}$:TReND:$Rab8a_{6\text{-}176}$ PDB code 6YX5**
**Wavelength (Å)**	0.966
**Resolution range**	48.02 - 2.14 (2.2 - 2.14)
**Space group**	P 3 2 1
**Unit cell**	142.3 142.3 76.6 90 90 120
**Total reflections**	980,292 (8,6871)
**Unique reflections**	49,091 (4,745)
**Multiplicity**	20.0 (18.3)
**Completeness (%)**	99.7 (97.0)
**Mean I/sigma(I)**	15.6 (0.93)
**Wilson B-factor**	43.5
**R-merge**	0.201 (2.43)
**R-meas**	0.206 (2.50)
**R-pim**	0.0458 (0.575)
**CC1/2**	0.999 (0.43)
**CC***	1 (0.775)
**Reflections used in refinement**	49,075 (4,744)
**Reflections used for R-free**	2,440 (227)
**R-work**	0.23 (0.35)
**R-free**	0.25 (0.36)
**CC(work)**	0.95 (0.57)
**CC(free)**	0.93 (0.56)
**Number of non-hydrogen atoms**	4,353
**macromolecules**	4,146
**ligands**	94
**solvent**	113
**Protein residues**	512
**RMS(bonds)**	0.013
**RMS(angles)**	1.7
**Ramachandran favored (%)**	97.2
**Ramachandran allowed (%)**	2.7
**Ramachandran outliers (%)**	0.00
**Rotamer outliers (%)**	2.7
**Clashscore**	2.9
**Average B-factor**	32.32
**macromolecules**	31.26
**ligands**	66.34
**solvent**	43.06
**Number of TLS groups**	2

Statistics for the highest-resolution shell are shown in parentheses.

Supplementary Table 3

Supplementary Table 3. Primers for cloning of bacterial plasmids

Primer		Sequence
**P1**	Fw	5' TAACTCGAGGGATCCGGG 3'
**P2**	Rv	5' GTGATGATGGTGGTGATGACCCATACGTTTTTTGATTT 3'
**P3**	Fw	5' CATCACCACCATCATCACTAACTCGAGGGATCCGGG 3'
**P4**	Rv	5' ACCCATACGTTTTTTGATTT 3'
**P13**	Fw	5'TCAGAAGTGAAACGCCGTAG 3'
**P17**	Rv	5' ATGCTTGTCGTCGTCATC 3'
**P15**	Fw	5’ GAAAAGGGTGCAGATGACGACGACAAGCATATGAGCATAATGGGGAGAAT 3’
**P16**	Rv	5’ GGCGCTACGGCGTTTCACTTCTGATCATTTTATCTTAATGGTTTGTC 3’
**P18**	Fw	5' GTTTAACTTTAAGAAGGAGATATACAAATGTTTGGTAGTTTATACAGTG 3'
**P19**	Rv	5' CTACGGCGTTTCACTTCTGATTACAACTTCTTGGCATTTTC 3'
**P20**	Fw	5’ TCAGAAGTGAAACGCCGTAG 3’
**P21**	Rv	5’TTGTATATCTCCTTCTTAAAGTTAAACAAAATTATTTCTGGTGAATTCC 3’
**P22**	Fw	5’ AAAGGAGAATCTTTATTTTCAGGGCTTTGGTAGTTTATACAGTGATGAGCGAG 3’
**P23**	Rv	5’ TCGAACTGCGGGTGGCTCCAAGACGCCATTTGTATATCTCCTTCTTAAAGTTAAACAAAATTATTTCTGGTGAATTC 3’
**P24**	Fw	5’ TTGCGTTTCTACAAACTCTTTTGT 3’
**P25**	Rv	5’ GGCCGCATCTAGAGGGCC 3’
**P26**	Fw	5’ GGCCCTCTAGATGCGGCCAAGAAACCAATTGTCCATATTG 3’
**P27**	Rv	5’ ACAAAAGAGTTTGTAGAAACGCAA 3’

Supplementary Table 4

Supplementary Table 4. Primers for cloning of mammalian plasmids

Primer		Sequence
**M1**	Fw	5' CTAGAGCTAGCACCATGCCGGAATATGACTATC 3'
**M2**	Rv	5' AATTATGGATCCCTCGAGTTAGTG 3'
**M5**	Fw	5’ TGATGCCTTTTGCGCGCTTGGTG 3’
**M6**	Rv	5’ TAAATCATTTCAGCGTAAATAGCAG 3’
**M7**	Fw	5’ TGATGCCTTTgctGCGCTTGGTG 3’
**M8**	Rv	5’ TAAATCATTTCTTTGTAAATTTGACG 3’
**M9**	Fw	5’ TGATGCCTTTtgtGCGCTTGGTG 3’
**M10**	Rv	5’ TAAATCATTTCTTTGTAAATTTGAC 3’

Supplementary Table 5

Supplementary Table 5. Primers for cloning of DrrA constructs in DrrA:Rab complexes study

Primer		Sequence
**wt-F**	Fw	5' TTTGGTAGTTTATACAGTGATGAGCGA 3'
**wt-R**	Rv	5' CATATGGCCCTGAAAATAAAGATTCTCATC 3'
**L197C-F**	Fw	5' TCGCTATGAAtgtGGAGAAGAGTTAC 3'
**L197C-R**	Rv	5' GGTAGAACAGGTTTTGATG 3'
**G198C-F**	Fw	5' CTATGAATTATGTGAAGAGTTACGAGATAAAATC 3'
**G198C-R**	Rv	5' CGAGGTAGAACAGGTTTTG 3'
**M169C-F**	Fw	5' TTTGTTTGGTTGTTTAAAAGACGGGATGGTAGC 3'
**M169C-R**	Rv	5' TCATCAGGCGTACCAATAAG 3'
**M174C-F**	Fw	5' AAAAGACGGGtgtGTAGCTGATGTTGAAG 3'
**M174C-R**	Rv	5' AACATACCAAACAAATCATC 3'
**V175C-F**	Fw	5' AGACGGGATGTGTGCTGATGTTGAAG 3'
**V175C-R**	Rv	5' TTTAACATACCAAACAAATCATC 3'
**N57A-F**	Fw	5' AAATGAGGGCgctGAAGTATCTCCATG 3'
**N57A-R**	Rv	5' TCTCGAATTATTTTACTTAGATTAG 3'
**Q63A-F**	Fw	5' ATCTCCATGGgctGAGTGGGAGAATG 3'
**Q63A-R**	Rv	5' ACTTCATTGCCCTCATTTTC 3'
**E64A-F**	Fw	5' TCCATGGCAGgctTGGGAGAATGG 3'
**E64A-R**	Rv	5' GATACTTCATTGCCCTCATTTTC 3'
**R70A-F**	Fw	5' GAATGGATTAgctCAAATTTACAAAGAAATGATTTATGATGCCTTTGACGC 3'
**R70A-R**	Rv	5' TCCCACTCCTGCCATGGA 3'
**Q71A-F**	Fw	5' TGGATTACGTgctATTTACAAAGAAATGATTTATGATGC 3'
**Q71A-R**	Rv	5' TTCTCCCACTCCTGCCAT 3'
**K74A-F**	Fw	5' TCAAATTTACgctGAAATGATTTATGATGCCTTTG 3'
**K74A-R**	Rv	5' CGTAATCCATTCTCCCAC 3'
**D79A-F**	Fw	5' AATGATTTATgctGCCTTTGACG 3'
**D79A-R**	Rv	5' TCTTTGTAAATTTGACGTAATC 3'
**R194A-F**	Fw	5' TGTTCTACCTgctTATGAATTAGGAGAAGAG 3'
**R194A-R**	Rv	5' GGTTTTGATGTTAAAATTGAC
**Y195A-F**	Fw	5' TCTACCTCGCgctGAATTAGGAGAAG 3'
**Y195A-R**	Rv	5' ACAGGTTTTGATGTTAAAATTG 3'
**E264A-F**	Fw	5' TTTATACAGTgctGATGGAGCTCATC 3'
**E264A-R**	Rv	5' TTAAATTCCTCACGCAATC 3'

Supplementary Table 6

Supplementary Table 6. Primers for cloning of DrrA constructs in MTS-experiments in H1299 cells

Primer		Sequence
**H1**	Fw	5' TAAGCGACTCTAGATCATAATCAGCCATAC 3'
**H2**	Rv	5' GCTTCCTCCTCCTCCCTTGTA 3'
**H3**	Fw	5' TTTGGTAGTTTATACAGTGATGAGCGA 3'
**H4**	Rv	5' GTATGGCTGATTATGATCTAGAGTCGCTTACAACTTCTTGGCATTTTCCAG 3'
**H5**	Fw	5' AAAATGAGTGTTAATGAAGAGCAATTTGGTAGTTTATACAGTGATGAGCGA 3'
**H6**	Rv	5' GTATGGCTGATTATGATCTAGAGTCGCTTATCATTTATAAGAAAGATATTCGGATAAATT
**H7**	Fw	5' CACAAGCCACTGAGTATAGTGCTTTGGCTGCCTTTGTTATTGTTAAAAA 3'
**H8**	Rv	5' TTTTTAACAATAACAAAGGCAGCCAAAGCACTATACTCAGTGGCTTGTG 3'
**H9**	Fw	5' TTAGCTGCTATTTACGCTGAAATGATTTATGATGCCTTTGACGC 3'
**H10**	Rv	5' AGCGTAAATAGCAGCTAATCCATTCTCCCACTCCTGCCA 3'
**H5**	Fw	5' AAAATGAGTGTTAATGAAGAGCAATTTGGTAGTTTATACAGTGATGAGCGA 3'
**H11**	Rv	5' TTCAAGCATGCTATTCGCGTTGTCCCACAACTTCTTGGCATTTTCCAGATTTTCAAT 3'
**H12**	Fw	5' AAATTGCTCTTCATTAACACTCATTTT 3'
**H13**	Rv	5' ATTGAAAATCTGGAAAATGCCAAGAAG 3'

Supplementary Note 1

Supplementary note on the analysis of mass-spectrometric crosslink data

In the mass-spectrometry based detection of crosslinks between $DrrA_{ATase}$ and Rab1b, we faced the challenge to find a very small portion of crosslinked peptides in an overwhelming background of unmodified peptides. Several improvement steps in the biochemical methodology finally made it possible to identify crosslinked peptides between the two target proteins. These peptides were detected using high-precision mass spectrometry coupled to the crosslink detection software Kojak [4, 5].

In order to account for human protein contamination, we appended a list of common contaminants to the $DrrA_{ATase}$/Rab1b search database and applied a target-decoy strategy based on reversed sequences to estimate the fraction of false positives in the output (see Methods section). For all of the inter-protein crosslink events, we identified 4,998 putative crosslinks and ~73% of these crosslinks bore either one or two decoy peptides. In the remaining ~27% of identifications only three could be attributed to the $DrrA_{ATase}$:Rab1b complex (Supplementary Table 1), whereas the others were crosslinks involving contaminant peptides.

As each identification comes with an assigned score, we then visualized the score distributions for decoy and target identifications to get a sense for the quality of the $DrrA_{ATase}$:Rab1b peptides in this context (Supplementary Figure 2). The plot clearly indicates that these crosslinks of interest fall in a score range, where the target distribution is

outperforming the decoy distribution. Additionally, the two best-scoring hits were assigned to the same crosslink site. We are aware of the fact that these spectra were acquired right after each other and thus likely stem from the same peptide, but given the notion that we did this analysis to get an initial idea, where a crosslink could happen, we took all this evidence and moved on to biochemically validate the finding.

Supplementary References

1. Winn MD*, et al.* Overview of the CCP4 suite and current developments. *Acta Crystallogr. D Biol. Crystallogr.* **67**, 235-242 (2011).

2. Laskowski RA, Swindells MB. LigPlot+: multiple ligand-protein interaction diagrams for drug discovery. *J. Chem. Inf. Model.* **51**, 2778-2786 (2011).

3. Hoopmann MR*, et al.* Kojak: efficient analysis of chemically cross-linked protein complexes. *J. Proteome Res.* **14**, 2190-2198 (2015).

4. Hoopmann MR, Mendoza L, Deutsch EW, Shteynberg D, Moritz RL. An Open Data Format for Visualization and Analysis of Cross-Linked Mass Spectrometry Results. *J. Am. Soc. Mass Spectrom.* **27**, 1728-1734 (2016).

5. Hoopmann MR*, et al.* Kojak: efficient analysis of chemically cross-linked protein complexes. *J. Proteome Res.* **14**, 2190-2198 (2015).
