# Supplementary material for: Rab1-AMPylation by Legionella DrrA is allosterically activated by Rab1
Source: Nat Commun. 2021 Jan 19;12:460. doi: 10.1038/s41467-020-20702-2 (PMC7815794; doi:10.1038/s41467-020-20702-2)
Supplement: Supplementary file 3 — Reporting Summary [file 41467_2020_20702_MOESM3_ESM.pdf]

nature research

Corresponding author(s): Kathrin Lang, Sabine Schneider, Aymelt Itzen

Last updated by author(s): Dec 11, 2020

Reporting Summary

Nature Research wishes to improve the reproducibility of the work that we publish. This form provides structure for consistency and transparency in reporting. For further information on Nature Research policies, see our Editorial Policies and the Editorial Policy Checklist.

Statistics

For all statistical analyses, confirm that the following items are present in the figure legend, table legend, main text, or Methods section.

n/a	Confirmed	
☐	☒	The exact sample size (n) for each experimental group/condition, given as a discrete number and unit of measurement
☐	☒	A statement on whether measurements were taken from distinct samples or whether the same sample was measured repeatedly
☐	☒	The statistical test(s) used AND whether they are one- or two-sided<br>*Only common tests should be described solely by name; describe more complex techniques in the Methods section.*
☒	☐	A description of all covariates tested
☒	☐	A description of any assumptions or corrections, such as tests of normality and adjustment for multiple comparisons
☐	☒	A full description of the statistical parameters including central tendency (e.g. means) or other basic estimates (e.g. regression coefficient) AND variation (e.g. standard deviation) or associated estimates of uncertainty (e.g. confidence intervals)
☐	☒	For null hypothesis testing, the test statistic (e.g. F, t, r) with confidence intervals, effect sizes, degrees of freedom and P value noted<br>*Give P values as exact values whenever suitable.*
☒	☐	For Bayesian analysis, information on the choice of priors and Markov chain Monte Carlo settings
☒	☐	For hierarchical and complex designs, identification of the appropriate level for tests and full reporting of outcomes
☐	☒	Estimates of effect sizes (e.g. Cohen's d, Pearson's r), indicating how they were calculated

Our web collection on statistics for biologists contains articles on many of the points above.

Software and code

Policy information about availability of computer code

Data collection	MTS (3-(4,5-dimethylthiazol-2-yl)-2,5-diphenyltetrazolium bromide) data was recorded with TECAN Spark (SparkControl™). Gel images were recorded with ChemoStar Touch v0.5.65 (Intas Science Imaging) and the scanner DCP-9022CDW (Brother International GmbH). Western blots were recorded with the western blot imager Fusion Pulse 6 (Vilber Lourmant). All kinetic data were recored with Hitachi F-2710 (FL Solutions 4.1). For DrrA:TReND:Rab complexes, high resolution mass spectra were recorded on an Agilent 6230 Series TOF mass-spectrometer coupled to an Agilent 1290 Infinity II LC system (Agilent MassHunter Data Aquisition, version B.06.01).
Data analysis	Crystallography data was analyzed with PHASER 2.8.2, PHENIX 1.18.2, COOT 0.8.9, REFMAC 5.8.0253, Pymol v1.8.6.2 was used for structure analysis. Kinetic study data were integrated with Origin 2019b. MTS data was integrated with Origin 2019b and Prism v5.0 (GraphPad). Total protein mass was calculated by deconvolution within the MS OpenLab ChemStation software Edition Rev. C.01.07 SR3 [465] (Agilent Technologies). For crosslink detection, mass spectrometry data were converted to mzML files using MSConvert in ProteoWizard. DrrA:TReND:Rab complexes masses were calculated by deconvolution within the MS Agilent MassHunter Qualitative Analysis (Agilent Technologies, version B.07.00). Crosslink searches were then performed with Kojak software version 1.5.5.

For manuscripts utilizing custom algorithms or software that are central to the research but not yet described in published literature, software must be made available to editors and reviewers. We strongly encourage code deposition in a community repository (e.g. GitHub). See the Nature Research guidelines for submitting code & software for further information.

Data

Policy information about availability of data

All manuscripts must include a data availability statement. This statement should provide the following information, where applicable:

- Accession codes, unique identifiers, or web links for publicly available datasets
- A list of figures that have associated raw data
- A description of any restrictions on data availability

All presented data are requested from the corresponding authors. The mass spectrometry proteomics data have been deposited at the ProteomeXchange Consortium (http://proteomecentral. proteomexchange.org) via the PRIDE partner repository, with data set identifier PXD019043. Crystallographic data for the DrrA-Rab8a complex have been deposited in the Protein Data Bank (https://www.ebi.ac.uk/pdbe/) under PDB accession code 6YX5. Hit selection and statistical analysis of Rab1b_Q67A_R69K-DrrA16-352 was performed using custom R scripts (https://github.com/higsch/crosslinkR).
For the following figures, associated raw data are available in the source data file: Figs 1c, 1i, 1g, 1h, 3a, 3b, 3d, 4d, 4e, 4f, 4g, 4h, 5b, 5c, 5d.
For the following supplementary figures, associated raw data are available in the source data file: 1a, 1b, 3a, 3b, 4, 5a, 5b, 10, 11c, 13, 14, 15, 17a, 17b, 17c.

Field-specific reporting

Please select the one below that is the best fit for your research. If you are not sure, read the appropriate sections before making your selection.

- [x] Life sciences
- [ ] Behavioural & social sciences
- [ ] Ecological, evolutionary & environmental sciences

For a reference copy of the document with all sections, see nature.com/documents/nr-reporting-summary-flat.pdf

Life sciences study design

All studies must disclose on these points even when the disclosure is negative.

Sample size	None of the statistical methods was used to predetermine sample size. To ensure data's reproducibility, three independent replicates were performed for each experiment.
Data exclusions	No data were excluded.
Replication	All attempt at replication are successful. Numbers of replications in different experiments can be found in the corresponding figure legends.
Randomization	Not relevant to the experimental design.
Blinding	Not relevant to the experimental design.

Reporting for specific materials, systems and methods

We require information from authors about some types of materials, experimental systems and methods used in many studies. Here, indicate whether each material, system or method listed is relevant to your study. If you are not sure if a list item applies to your research, read the appropriate section before selecting a response.

Materials & experimental systems

n/a	Involved in the study	
[ ]	[x]	Antibodies
[ ]	[x]	Eukaryotic cell lines
[x]	[ ]	Palaeontology and archaeology
[x]	[ ]	Animals and other organisms
[x]	[ ]	Human research participants
[x]	[ ]	Clinical data
[x]	[ ]	Dual use research of concern

Methods

n/a	Involved in the study	
[x]	[ ]	ChIP-seq
[ ]	[x]	Flow cytometry
[x]	[ ]	MRI-based neuroimaging

Antibodies

Antibodies used	Mouse monoclonal antibody Anti-His6-Peroxidase (11 965 085 001; Roche Applied Science (Penzberg, Germany); 1:5000 dilution); monoclonal antibody StrepMAB-Classic, HRP conjugate (2-1509-001; IBA GmbH (Göttingen, Germany); 1:5000 dilution); mouse monoclonal antibody anti GFP (B-2) (sc-9996; Santa Cruz Biotechnology (Dallas, USA); 1:1000 dilution); Polyclonal Goat anti-Mouse IgG (H+L) Secondary Antibody, HRP (32430; Invitrogen - Thermo Fisher Scientific (Waltham, USA); 1:500 dilution)
Validation	All antibodies were sourced commercially and respective controls were included in each experiment.<br>Mouse monoclonal antibody Anti-His6-Peroxidase (validated for WB, ELISA; tag specific; Roche; https://www.sigmaaldrich.com/catalog/product/roche/11965085001 (last access 04.12.2020); validated according to the manufacturer by Western blot using

extracts from cell line expressing a recombinant His6-tagged protein & according to the distributors homepage, product meets standard MQ100 (ISO9001, certificate of quality or certificate of analysis available, testing performed according to established protocol).
Monoclonal antibody StrepMAB-Classic, HRP conjugate (validated for WB; tag specific; IBA GmbH; https://www.iba-lifesciences.com/details/product/68.html (last access 04.12.2020); according to the distributors homepage, product meets ISO9001:2015.).
Mouse monoclonal antibody anti GFP (B-2) (validated for WB, IP, Immunofluorescence, ELISA; tag specific; Santa Cruz Biotechnology; https://www.scbt.com/de/p/gfp-antibody-b-2 (last access 04.12.2020); sc-9996 was tested by manufacturer in immunofluorescence staining of methanol-fixed COS cells transfected with GFP fusion protein showing cytoplasmic staining &
sc-9996-AF680 was further testes in direct near-infrared western blot analysis of GFP expression in human recombinant GFP fusion protein.).
Polyclonal Goat anti-Mouse IgG (H+L) Secondary Antibody, HRP (validated for WB, ELISA; mouse specific; Invitrogen; https://www.thermofisher.com/antibody/product/Goat-anti-Mouse-IgG-H-L-Secondary-Antibody-Polyclonal/32430 (last access 04.12.2020))

Eukaryotic cell lines

Policy information about cell lines

Cell line source(s)	H1299 cell line (ATCC® CRL-5803™), HEK293T cell line HEK293T cell line (The European Collection of Authenticated Cell Cultures (ECACC,Cat# 12022001, RRID:CVCL_0063))
Authentication	Cell lines have been provided and authenticated by the provider. No additional authentication has been performed in our labs.
Mycoplasma contamination	Cells were free of mycoplasma contamination.
Commonly misidentified lines (See ICLAC register)	Commonly misidentified cell lines were not used in the present study.

Flow Cytometry

Plots

Confirm that:

- [x] The axis labels state the marker and fluorochrome used (e.g. CD4-FITC).
- [x] The axis scales are clearly visible. Include numbers along axes only for bottom left plot of group (a 'group' is an analysis of identical markers).
- [x] All plots are contour plots with outliers or pseudocolor plots.
- [x] A numerical value for number of cells or percentage (with statistics) is provided.

Methodology

Sample preparation	Supernatant was removed. H1299 cells were first washed with DPBS (gibco), then trypsinized using trypsin/EDTA (gibco). After centrifugation (5 min at 200 g and 4°C) cells were washed two more times with DPBS, and kept in 1.5ml DPBS for flow cytometric analysis. A total of 40000 cells were collected per sample and BD FACS Diva software (BD Biosciences) was used for data evaluation.
Instrument	BD FACS Aria Fusion, Special Order System
Software	BD FACS Diva, Version 8.0.1
Cell population abundance	The H1299 cells can be divided into two sub-populations, GFP-negative cells and GFP-positive cells. The GFP-positive cell can be easily discriminated and separated from the GFP- negative cells. To confirm there is no contamination in the sorted GFP-positive population, microscopy inspection was performed.
Gating strategy	Detailed gating strategy is stated Supplementary Information. Briefly, dead H1299 cells and debris were first excluded by applying the Side scatter area (SSC-A) versus forward scatter area (FSC-A) density plot. Following, the FSC-H versus FSC-W plot and the SSC-H versus SSC-W plot were applied to ensure the selection of single cells. The last FITC-A versus FSC-A plot was applied to separate the GFP-postive cells from GFP-negative cells. The boundary between the GFP-postive cells from GFP-negative cells were defined by comparing non-transfected H1299 cells (which has no GFP-postive cells for further collection) and GFP-transfected H1299 cells.

- [x] Tick this box to confirm that a figure exemplifying the gating strategy is provided in the Supplementary Information.
